# Technique and device specific diffusion-weighted imaging detected ischemic lesions and occlusion outcomes in endovascular treatment of unruptured aneurysms

**DOI:** 10.1007/s00701-026-06934-z

**Published:** 2026-06-04

**Authors:** Rahul Raj, Jussi Numminen, Mika Niemelä, Miikka Korja

**Affiliations:** 1 Department of Neurosurgery, Helsinki University Hospital and University of Helsinki, Helsinki, P.O. Box 320, Haartmaninkatu 4, FI-00029 Helsinki, Finland; 2 Department of Radiology, Helsinki University Hospital and University of Helsinki, Helsinki, Finland

**Keywords:** Intracranial aneurysm, MRI, Complications, Endovascular neurointervention, Outcome

## Abstract

**Background:**

This prospective study evaluated 6-month aneurysm occlusion and diffusion-weighted imaging (DWI) lesion rates following endovascular treatment of unruptured intracranial aneurysms (UIAs). We hypothesized that complex techniques would yield better occlusion but more DWI lesions.

**Methods:**

We conducted a single-center, prospective cohort study of consecutive patients treated for UIAs with 6-month angiographic follow-up. Treatment technique was categorized as complex (flow diversion, stent-assisted coiling [SAC], or balloon-assisted coiling [BAC]) or non-complex (simple coiling, Trenza-assisted coiling, or intrasaccular device placement). Aneurysm morphology was classified as complex and non-complex according to a Delphi statement. Primary outcomes were adequate occlusion (complete or neck residual) at 6 months and postprocedural DWI lesions, assessed by MRI.

**Results:**

In 113 patients, complex treatment was used in 96% (*n* = 25/26) of complex aneurysms, whereas 57% (*n* = 53/93) of non-complex aneurysms underwent non-complex treatment (*p* < 0.001). There were no differences in occlusion rates (90% vs. 81%, *p* = 0.181) or DWI rates (56% vs. 69%, *p* = 0.124) between non-complex and complex treatments. Aneurysm complexity associated with lower occlusion rates (67% vs. 90%, *p* = 0.012) but not with DWI lesion rates (58% vs. 65%, *p* = 0.524). Notably, individual techniques demonstrated greater variation in DWI rates (42–100%) than in occlusion rates (70–100%).

**Conclusion:**

Occlusion rates at 6 months were high across techniques, but DWI lesion rates varied substantially between individual techniques. The ischemic burden appears to be driven by the specific endovascular technique rather than by broad categorizations of procedural complexity. Thus, careful technique selection is critical to minimize ischemic risk while appropriately addressing aneurysm complexity.

**Supplementary Information:**

The online version contains supplementary material available at 10.1007/s00701-026-06934-z.

## Introduction

The management of unruptured intracranial aneurysms (IA) has evolved rapidly over the past two decades, shifting from primarily surgical approaches to endovascular techniques, driven by advances in endovascular techniques and devices [7, 32, 37]. The primary objective of UIA treatment is to achieve durable aneurysm occlusion without complications, thereby preventing the future risk of subarachnoid hemorrhage (SAH) and increasing quality-adjusted life years. Modern endovascular techniques and devices, including balloon-assisted coiling (BAC), stent-assisted coiling (SAC), flow diversion (FD), and intrasaccular devices, have expanded treatment options, particularly for complex and wide-necked aneurysms, with occlusion rates ranging from 70–90% [28, 31, 33].

Despite their efficacy, endovascular procedures carry a risk of thromboembolic complications, leading to symptomatic brain ischemia in approximately 5% of cases [1, 17, 19, 27]. A growing concern is the high incidence of silent ischemic lesions detected by diffusion-weighted imaging (DWI) on magnetic resonance imaging (MRI), reported in 37–62% of patients [15]. Although often asymptomatic in the short term, these DWI-detected lesions raise concern about potential long-term consequences. Evidence from population-based studies links spontaneous silent brain infarcts to cognitive decline and dementia [2, 3, 35, 36], though it remains uncertain whether procedurally induced DWI lesions, which differ in pathogenesis, size, and distribution, carry the same long-term risk. Importantly, it has been reported that DWI lesion rates vary substantially by technique and devices, with flow diversion (62%), SAC (48%) and BAC (37%) showing highest rates of ischemic lesions [15].

Comparisons of DWI lesions and occlusion outcomes by various techniques remain limited in prospective cohorts. Such data would be important for optimizing technique and device selection based on aneurysm and patient characteristics, thus balancing ischemic risk against durable occlusion. This study aimed to prospectively evaluate technique-specific and device-specific DWI-detected ischemic lesions and 6-month occlusion rates in endovascularly treated UIAs. We hypothesized that complex endovascular techniques would achieve superior aneurysm occlusion rates at the cost of increased ischemic events, as evidenced by DWI lesions.

## Methods and analysis

### Study setting and design

We conducted a prospective observational cohort study of consecutive patients treated for UIAs at Helsinki University Hospital (catchment area 2.2 million) between December 2022 and August 2024. Patient population, treatment protocols, and institutional setting have been previously described [30]. Eligible patients included those undergoing endovascular treatment for UIAs, excluding those with aneurysms related to arteriovenous malformations or moyamoya disease. Patients treated with parent artery occlusion were not included as our analysis focused on device-specific endovascular techniques. Our institutional treatment policy favors microsurgical clipping for middle cerebral artery (MCA) aneurysms and endovascular approaches for other locations [7].

We obtained informed consent from all study patients. The study was approved by the HUS Regional Committee on Medical Research Ethics (HUS/16731/2022). The study was registered at ClinicalTrials.gov, NCT06147102. The reporting of the study was in accordance to the STrengthening the Reporting of OBservational studies in Epidemiology guidelines [13].

### Endovascular treatments

We defined treatment complexity a priori according to the endovascular technique used. Flow diversion, SAC, and BAC were classified as complex techniques because they require an additional temporary (BAC) or permanent (SAC, flow diversion) device in the parent vessel and have been associated with greater procedural complexity and thromboembolic risk [1, 15]. Simple coiling, Trenza-assisted coiling, and intrasaccular device placement were classified as non-complex techniques because they involve only intrasaccular device placement, without an adjunctive parent-vessel device.

Patients scheduled for simple coiling or BAC of narrow-neck aneurysms typically did not receive pretreatment antiplatelet therapy, whereas those with broad-neck aneurysms planned for coiling received antiplatelets in anticipation of potential bailout stenting. All patients undergoing planned SAC, flow diversion (FD), or intrasaccular device placement received dual antiplatelet therapy (DAPT) pre-procedure. After the procedure, patients treated with simple coiling, BAC, or intrasaccular devices received single antiplatelet therapy (SAPT) for 1 month. Those treated with SAC received DAPT for 3 months, followed by SAPT for an additional 3 months. Patients treated with FD received DAPT for 6 months. In case of bailout stenting (*n* = 2), a tirofiban bolus and infusion was administered, followed by DAPT loading.

### Outcomes and definitions

Adequate occlusion was defined as follows: for coiled aneurysms, modified Raymond-Roy Occlusion Classification (RROC) I (complete obliteration) or II (residual neck) [22]; for intrasaccular devices, complete obliteration, filling of the recess, or aneurysm neck filling [8, 9]; and for flow diversion, complete obliteration or residual filling confined to the neck region without extending beyond its width [18].

Brain MRIs were independently evaluated by board-certified neuroradiologists and two study authors (R.R., M.K.). Discrepancies were resolved by a third author (J.N.), a neuroradiologist with over 20 years of experience. Inter-rater agreement was high (Cohen’s kappa = 0.81, 95% CI: 0.71–0.89) [30]. DWI lesions were defined as any DWI lesions ≥ 1 mm on postprocedural brain MRI, performed within 3 days of treatment using 1.5 T or 3.0 T scanners. MRI sequences included DWI, non-contrast T1 and T2, apparent diffusion coefficient map, and fluid-attenuated inversion recovery (FLAIR). The DWI sequence comprised a multisection, single-shot, spin-echo echo-planar imaging sequence. Diffusion gradients were applied in each of the x, y, and z directions with 2b values (0 and 1000 SE/mm2). Imaging parameters included a field of view of 245 × 245 mm, 178/169 acquisition matrix, TE of 66 ms, section thickness of 3 mm, and an intersection gap of 0.

Clinical outcomes were assessed as changes in functional status from baseline to 3-month follow-up, measured using the modified Rankin Scale (mRS; 0 = no symptoms, 6 = death) [6].

### Statistical analysis

We report summary group statistics with continuous variables as mean values (normally distributed data) or medians (non-parametric data) with standard deviations (SDs) and interquartile ranges (IQRs). We report categorical variables as relative frequencies. Binary endpoints were compared between groups using the chi-square (Fisher’s exact test when numbers were low), and continuous endpoints were compared between groups using the Wilcoxon rank-sum test.

To evaluate the balance between aneurysm occlusion and ischemic risk across endovascular technique complexity and individual devices, we employed a quadrant analysis. This method categorized each technique device into one of four quadrants based on its 6-month adequate occlusion rate and postprocedural MRI-DWI lesion rate, relative to the population’s mean values for these outcomes. Outcomes were classified as follows: (1) high occlusion, low ischemia (occlusion rate ≥ mean adequate occlusion rate, DWI lesion rate < mean DWI lesion rate); (2) high occlusion, high ischemia (occlusion rate ≥ mean adequate occlusion rate, DWI lesion rate ≥ mean DWI lesion rate); (3) low occlusion, low ischemia (occlusion rate < mean adequate occlusion rate, DWI lesion rate < mean DWI lesion rate); and (4) low occlusion, high ischemia (occlusion rate < mean adequate occlusion rate, DWI lesion rate ≥ mean DWI lesion rate).

We analyzed the risk of any DWI lesion and the risk of ≥ 6 DWI lesions according to the definition of Kang and colleagues [19]. We conducted subgroup analyses according to aneurysm complexity and treatment complexity. Consistent with a recent Delphi study, complex aneurysms were defined by the presence of at least one of the following characteristics: size ≥ 25 mm, dome-to-neck ratio < 1.0, or a collateral branch arising from the aneurysm sac [12].

Participants with missing data were excluded from the analysis. All statistical analyses were performed using Stata, version 19. 

### Ethics approval and consent to participate

The study was conducted in accordance with the Helsinki Declaration and registered on ClinicalTrials.gov (NCT06147102). The study was approved by the HUS Regional Committee on Medical Research Ethics (HUS/16731/2022).

### Consent to participate and consent to publish

Informed consent was obtained from all individual participants included in the study.

### Competing interests

The authors declare no competing interests.

## Results

### Baseline characteristics

**Fig. 1:**
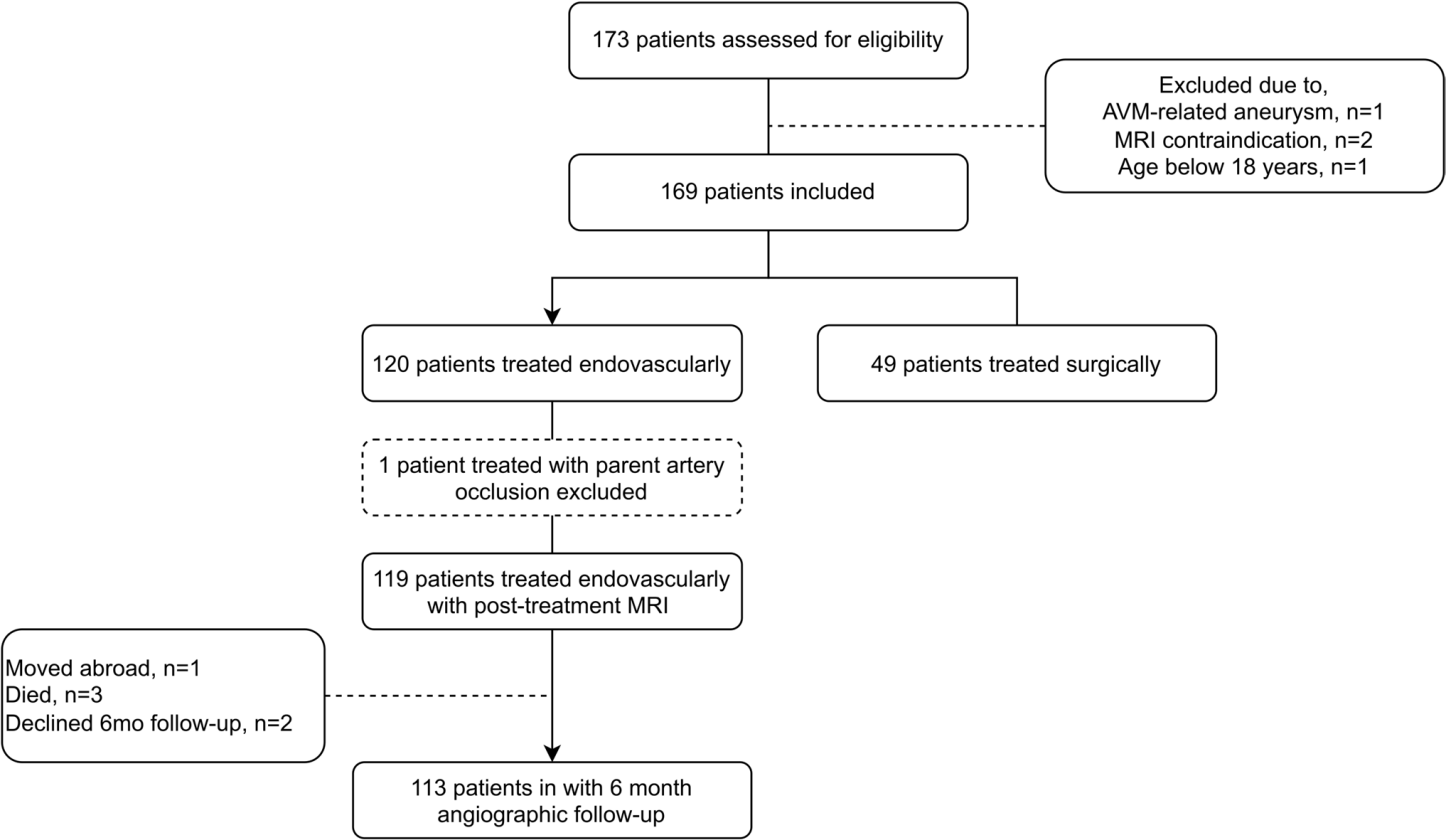
Flow chart. Abbreviations: AVM, arteriovenous malformations; MRI, magnetic resonance imaging

Of 120 patients treated endovascularly for UIAs, one was excluded due to treatment with parent artery occlusion. Six patients were lost to follow-up: death (*n* = 3), declined follow-up (*n* = 2), and moved abroad (*n* = 1). Of the three patients that died before angiographic follow-up, one died due to intraprocedural perforation during coiling (2.9 mm non-complex ACOM aneurysm), one died due to post-procedural hemorrhage 2 days after flow diversion (28 mm complex vertebral artery aneurysm), and one died due to non-aneurysmal or treatment related causes 5 months after flow diversion (19 mm complex basilar tip aneurysm). Thus, of 119 patients, 113 had 6-month angiographic follow-up (Fig. 1).

The median age was 58 years; 69% were female, 63% had hypertension, 31% were current smokers, and 21% had previous aneurysmal SAH. The most common aneurysm locations were the internal carotid artery (ICA, 48%) and anterior cerebral artery (ACA, 33%) (Table 1). Complex aneurysm morphology was present in 21% of cases.

**Table 1:** Patient and aneurysm characteristics by aneurysm occlusion grade

Variable	All patients (*N* = 113)	Adequate Occlusion (*N* = 96)	Inadequate Occlusion (*N* = 17)	*P*-Value
Patient baseline characteristics				
Age (years), median (IQR)	58 (53–64)	59 (54–65)	56 (51–61)	0.366
Sex				0.777
Female	78 (69)	67 (70)	11 (65)	
Male	35 (31)	29 (30)	6 (35)	
BMI, median (IQR)	27 (24–30)	27 (24–30)	26 (24.5–30.5)	0.961
Hypertension				0.862
No	42 (37)	36 (38)	6 (35)	
Yes	71 (63)	60 (63)	11 (65)	
Systolic BP, median (IQR)	141 (131–154)	141 (128–153)	142 (132–156)	0.435
Diastolic BP, median (IQR)	89 (84–98)	90 (84–98)	86 (82–95)	0.413
Diabetes mellitus				0.065
No	105 (93)	91 (95)	14 (82)	
Yes	8 (7)	5 (5)	3 (18)	
Previous SAH	24 (21)	23 (24)	1 (6)	0.116
Smoking status				0.987
Never	45 (40)	38 (40)	7 (41)	
Current smoker	35 (31)	30 (31)	5 (29)	
Previous smoker	33 (29)	28 (29)	5 (29)	
Aneurysm characteristics				
Target aneurysm is a retreatment	17 (15)	14 (15)	3 (18)	0.745
Modality of primary treatment				
Endovascular	15 (13)	12 (13)	3 (18)	0.486
Surgical	2 (2)	2 (2)	0 (0)	
Aneurysm location				0.591
ICA segments	54 (48)	46 (48)	8 (47)	
Anterior cerebral artery	37 (33)	32 (33)	5 (29)	
Middle cerebral artery	5 (4)	5 (5)	0 (0)	
Posterior circulation	17 (15)	13 (14)	4 (24)	
Complex aneurysm*	24 (21)	16 (17)	8 (47)	0.005
Aneurysm largest diameter (mm), median (IQR)	5.7 (4.2–8.8)	5.6 (4.0–8.6)	8.5 (4.6–12.7)	0.074
Dome-to-neck ratio, median (IQR)	1.4 (1.2–1.8)	1.5 (1.2–1.8)	1.4 (1.0–2.0)	0.936
Aspect ratio, median (IQR)	1.4 (1.0–1.9)	1.4 (1.0–1.9)	1.4 (1.0–2.1)	0.679

*BMI* Body mass index, *BP *Blood pressure, *IQR* Interquartile range, *mm* Millimeters, *mo *Month, *mRS* modified Rankin Scale, *SAH *Subarachnoid hemorrhage

*Defined as aneurysm ≥25 mm in size, dome-to-neck ratio <1.0, collateral branch arising from the aneurysm sac

Postprocedural MRI at median 2 days (IQR 2–2) showed any DWI lesions in 75 of 119 UIA treated patients (63.0%), and in 70 of 113 patients (62.0%) with a 6-month follow-up. Patients with DWI lesions had larger aneurysms (6.4 mm vs. 5.1 mm, *p* = 0.059) and higher dome-to-neck ratios (1.5 vs. 1.3, *p* = 0.026) (Table 2). Among 75 patients with DWI lesions, 17% (*n* = 13/75) developed new neurological deficits compared to one patient without DWI lesions (*n* = 1/44, *p* = 0.016). Of these deficits, 57% (*n* = 8/14) were transient and 43% (*n* = 6/14) were permanent. The presence of a DWI (17% vs. 2%, *p* = 0.014) or ≥ 6 DWI lesions (29% vs. 9%, *p* = 0.015) significantly associated with the risk of developing a new neurological symptom.

**Table 2:** Patient and aneurysm characteristics by MRI-DWI status

Variable	All patients (*N* = 119)	MRI-DWI lesion (*N* = 75)	No MRI-DWI lesion (*N* = 44)	*P*-Value
Patient baseline characteristics				
Age (years), median (IQR)	58 (52–64)	59 (54–65)	57 (48–63)	0.078
Sex				0.896
Female	82 (69)	52 (69)	30 (68)	
Male	37 (31)	23 (31)	14 (32)	
BMI, median (IQR)	27 (24–30)	27 (23–29)	27 (24–30)	0.554
Hypertension				0.301
No	45 (38)	31 (41)	14 (32)	
Yes	74 (62)	44 (59)	30 (68)	
Systolic BP, median (IQR)	140 (130–153)	143 (131–155)	136 (126–147)	0.139
Diastolic BP, median (IQR)	88 (83–98)	90 (84–98)	87 (82–98)	0.341
Diabetes mellitus				0.468
No	111 (93)	69 (92)	42 (95)	
Yes	8 (7)	6 (8)	2 (5)	
Previous SAH	24 (20)	17 (23)	7 (16)	0.375
Smoking status				0.614
Never	47 (40)	32 (43)	15 (34)	
Current smoker	39 (33)	24 (32)	15 (34)	
Previous smoker	33 (28)	19 (25)	14 (32)	
Aneurysm characteristics				
Target aneurysm is a retreatment	17 (14)	14 (19)	3 (7)	0.104
Modality of primary treatment				0.486
Endovascular	15 (13)	12 (16)	3 (7)	
Surgical	2 (2)	2 (3)	0 (0)	
Aneurysm location				0.254
ICA segments	55 (46)	38 (51)	17 (39)	
Anterior cerebral artery	40 (34)	26 (35)	14 (32)	
Middle cerebral artery	5 (4)	2 (3)	3 (7)	
Posterior circulation	19 (16)	9 (12)	10 (23)	
Complex aneurysm†	26 (22)	15 (20)	11 (25)	0.524
Aneurysm largest diameter (mm), median (IQR)	5.7 (4.1–8.8)	6.4 (4.4–9.3)	5.1 (3.8–7.4)	0.059
Dome-to-neck ratio, median (IQR)	1.5 (1.2–1.8)	1.5 (1.2–1.9)	1.3 (1.1–1.6)	0.026
Aspect ratio, median (IQR)	1.4 (1.0–1.9)	1.5 (1.0–2.0)	1.3 (1.0–1.7)	0.238

*BMI* Body mass index, *BP* Blood pressure, *IQR* Interquartile range, *mm* millimeters, *mo* month, *SAH* Subarachnoid hemorrhage

*Ruptured or unruptured aneurysm

†Defined as aneurysm ≥25 mm in size, dome-to-neck ratio <1.0, collateral branch arising from the aneurysm sac

At median angiographic follow-up of 6.6 months (IQR 6.0–8.0), 85% achieved adequate occlusion. Inadequately occluded aneurysms were more frequently complex (47% vs. 17%, *p* = 0.005) and larger (median 8.5 mm vs. 5.6 mm, *p* = 0.074) (Table 1). Two aneurysms were retreated using flow diversion after 5.7 months (4.1 mm ICA aneurysm initially treated by coiling) and 9.4 months (11.5 mm ICA aneurysm initially treated by Trenza-assisted coiling) from the index treatment.

Of the patients alive at the 6 month clinical follow-up (97.5%, *n* = 116/119), all had an mRS of 0–1, with 111 (95.7%) showing no deterioration from baseline and 5 patients (4.3%) deteriorating from mRS 0 to mRS 1.

### Endovascular treatment techniques and devices

Treatment selection generally matched aneurysm complexity: 96% (*n* = 25/26) of complex aneurysms underwent complex treatment, while 57% (*n* = 53/93) of non-complex aneurysms underwent non-complex treatment (*p* < 0.001).

The complex treatment group was characterized by younger patient age (median 56 vs. 61 years, *p* = 0.062), a higher proportion of cases being retreatments (26% vs. 0%, *p* < 0.001), a greater prevalence of ICA aneurysms (66% vs. 22%, *p* < 0.001), and larger aneurysm size (median 6.1 vs. 5.2 mm, *p* = 0.032) compared to the non-complex group (Table 3).

**Table 3:** Patient and aneurysm characteristics by treatment complexity

Variable	All patients (*N* = 119)	Non-complex treatment (*N* = 54)	Complex treatment (*N* = 65)	*P*-Value
Patient baseline characteristics				
Age (years), median (IQR)	58 (52–64)	61 (56–64)	56 (50–63)	0.062
Sex				0.753
Female	82 (69)	38 (70)	44 (68)	
Male	37 (31)	16 (30)	21 (32)	
BMI, median (IQR)	27 (24–30)	27 (24–30)	27 (24–31)	0.944
Hypertension				0.549
No	45 (38)	22 (41)	23 (35)	
Yes	74 (62)	32 (59)	42 (65)	
Systolic BP, median (IQR)	140 (130–153)	139 (127–155)	140 (131–153)	0.865
Diastolic BP, median (IQR)	88 (83–98)	90 (84–99)	87 (82–97)	0.517
Diabetes mellitus				0.786
No	111 (93)	50 (93)	61 (94)	
Yes	8 (7)	4 (7)	4 (6)	
Previous SAH	24 (20)	7 (13)	17 (26)	0.074
Smoking status				0.704
Never	47 (40)	20 (37)	27 (42)	
Current smoker	39 (33)	17 (31)	22 (34)	
Previous smoker	33 (28)	17 (31)	16 (25)	
Aneurysm characteristics				
Target aneurysm is a retreatment	17 (14)	0 (0)	17 (26)	< 0.001
Modality of primary treatment				-
Endovascular	15 (13)	0 (0)	15 (23)	
Surgical	2 (2)	0 (0)	2 (3)	
Aneurysm location				< 0.001
ICA segments	55 (46)	12 (22)	43 (66)	
Anterior cerebral artery	40 (34)	29 (54)	11 (17)	
Middle cerebral artery	5 (4)	4 (7)	1 (2)	
Posterior circulation	19 (16)	9 (17)	10 (15)	
Complex aneurysm†	26 (22)	1 (2)	25 (38)	< 0.001
Aneurysm largest diameter (mm), median (IQR)	5.7 (4.1–8.8)	5.2 (3.9–8.0)	6.1 (4.6–14.1)	0.032
Dome-to-neck ratio, median (IQR)	1.5 (1.2–1.8)	1.5 (1.2–1.8)	1.4 (1.1–1.8)	0.339
Aspect ratio, median (IQR)	1.4 (1.0–1.9)	1.5 (1.1–1.9)	1.4 (0.9–1.9)	0.339

Treatment complexity was defined a priori. Complex treatment included flow diversion, stent-assisted coiling, and balloon-assisted coiling; non-complex treatment included simple coiling, Trenza-assisted coiling, and intrasaccular device placement

*BMI* Body mass index, *BP* Blood pressure, *IQR* Interquartile range, *mm* millimeters, *mo* month, *SAH* Subarachnoid hemorrhage

*Ruptured or unruptured aneurysm

†Defined as aneurysm ≥25 mm in size, dome-to-neck ratio <1.0, collateral branch arising from the aneurysm sac

The most frequent device was flow diversion (43%), followed by simple coiling (20%), Trenza-assisted coiling (14%) and intrasaccular devices (12%), SAC (8%) and BAC (3%). Flow diversion was predominantly used for ICA aneurysms, while coiling and intrasaccular devices were distributed across locations (Supplementary Table 1).

Aneurysms treated by Trenza-coiling (median 8.7 mm) and flow diversion (median 7.6 mm) were significantly larger in diameter than aneurysms treated with other devices (median 4.1–5.5 mm, *p* < 0.001). Median procedure duration ranged from 69 min for coiling to 108 min for SAC. All flow-diverted patients, 80% of SAC patients and 88% of Trenza-coiling received preprocedural DAPT, whereas 25% of coiling patients and 43% of intrasaccular device patients received preprocedural DAPT (Supplementary Table 2).

### Aneurysm occlusion and MRI-DWI lesions

Analysis of DWI lesion rates in all 119 treated patients revealed no significant association with either aneurysm complexity (58% vs. 65%, *p* = 0.524) or treatment complexity (69% vs. 56%, *p* = 0.124, Table 4). Similarly, the incidence of a high DWI burden (≥ 6 lesions), which occurred in 17 patients (14%), did not differ between complex and non-complex treatment strategies (17% vs. 11%, *p* = 0.367). In contrast to these findings, an analysis of the 113 patients with 6-month follow-up showed that aneurysm complexity was associated with significantly lower adequate occlusion rates (67% vs. 90%, *p* = 0.012). Notably, individual devices demonstrated substantially greater variation in DWI rates (42–100%) than in occlusion rates (70–100%).

**Table 4:** Aneurysm occlusion and MRI-DWI lesions by endovascular treatment technique

Treatment technique	Patients (n)†	DWI Lesions (%) (95% CI)	≥ 6 DWI lesions (%) (95% CI)	Adequate occlusion (%) (95% CI)
All aneurysms	**119**	**63 (54–72)**	**14 (8–21)**	**85 (78–92)**
Non-complex treatment	54	56 (42–69)	11 (3–20)	90 (82–99)
Coiling	24	42 (21–62)	4 (− 4–12)	91 (78–104)
Trenza-coiling	16	69 (45–93)	31 (8–55)	81 (61–101)
Intrasaccular device	14	64 (38–91)	0 (*)	100 (*)
Complex treatment	65	69 (58–81)	17 (8–26)	81 (71–91)
SAC	10	90 (70–110)	40 (8–72)	70 (40–100)
BAC	4	100 (*)	50 (− 7–107)	100 (*)
Flow diversion	51	63 (49–76)	10 (2–18)	82 (71–93)
Complex aneurysms	**26**	**58 (37–78)**	**8 (− 3–19)**	**67 (46–87)**
Non-complex treatment	1	0 (*)	0 (*)	100 (*)
Coiling	1	0 (*)	0 (*)	100 (*)
Complex treatment	25	60 (39–81)	8 (− 3–19)	65 (44–86)
SAC	4	75 (24–127)	25 (− 27–77)	50 (− 10–110)
Flow diversion	21	57 (34–80)	5 (− 5–15)	68 (46–91)
Non-complex aneurysms	**93**	**65 (55–74)**	**16 (9–24)**	**90 (84–96)**
Non-complex treatment	53	57 (43–70)	11 (3–20)	90 (81–99)
Coiling	23	44 (23–65)	4 (− 4–13)	90 (76–104)
Trenza-coiling	16	69 (45–93)	31 (8–55)	81 (61–101)
Intrasaccular device	14	64 (38–91)	0 (*)	100 (*)
Complex treatment	40	75 (61–89)	23 (9–36)	90 (81–100)
SAC	6	100 (*)	50 (6–94)	83 (50–117)
BAC	4	100 (*)	50 (− 7–107)	100 (*)
Flow diversion	30	67 (49–84)	13 (1–26)	90 (79–101)

Treatment complexity was defined a priori. Complex treatment included flow diversion, stent-assisted coiling, and balloon-assisted coiling; non-complex treatment included simple coiling, Trenza-assisted coiling, and intrasaccular device placement

*95% CI not calculable or omitted

†DWI lesion rates were calculated for all 119 patients undergoing treatment and occlusion rates were calculated for 113 patients with available 6 month angiographic follow-up

*BAC* Balloon Assisted Coiling, *SAC* Stent Assisted Coiling

**Fig. 2:**
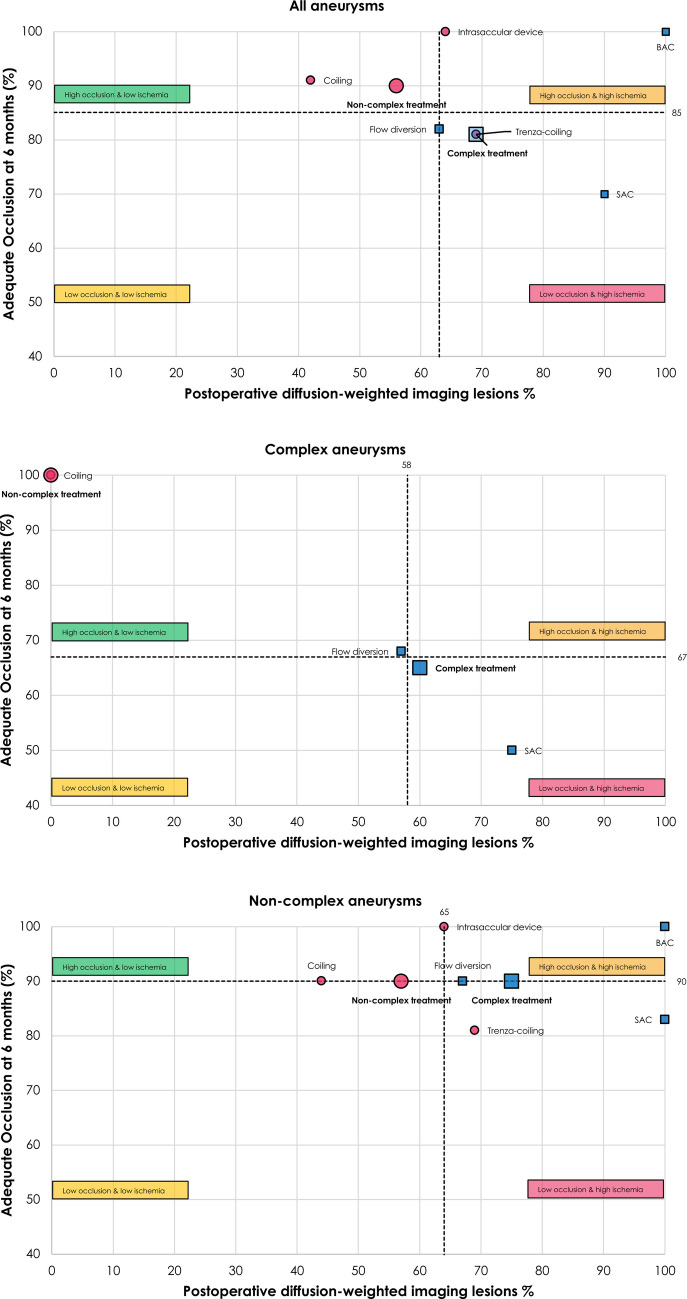
Distribution of endovascular techniques by 6-month occlusion and DWI lesion rates across all aneurysms (top), complex aneurysms (middle), and non-complex aneurysms (bottom). Quadrants represent optimal (green), intermediate (yellow), and suboptimal (pink) risk–benefit profiles based on means (dotted line)

A quadrant analysis was performed to visualize the risk–benefit profiles of different treatment strategies (Fig. 2). When assessing all aneurysms, non-complex treatment demonstrated a superior profile, achieving high occlusion with low ischemia (90% occlusion, 56% DWI lesions), while complex treatment was associated with low occlusion and high ischemia (81% occlusion, 69% DWI lesions). For complex aneurysms, a direct comparison of treatment strategies was limited as only one patient received non-complex treatment. Within complex techniques, flow diversion was more favorable (68% occlusion, 57% DWI lesions) compared to stent-assisted coiling (50% occlusion, 75% DWI lesions), though these differences were not statistically significant (*p* = 0.626 for DWI, *p* = 0.589 for occlusion). Among non-complex aneurysms, both strategies yielded high occlusion rates (90%), but non-complex treatment was associated with a lower ischemic burden (57% DWI lesions) than complex treatment (75% DWI lesions), a difference that trended toward significance (*p* = 0.066). Specifically, simple coiling demonstrated an optimal profile (90% occlusion, 44% DWI lesions), whereas flow diversion resulted in a high rate of both occlusion and ischemia (90% occlusion, 67% DWI lesions, *p* = 0.091).

A high DWI burden (≥ 6 lesions) was observed in 14% of all patients (Table 4). The rate did not differ significantly by overall treatment complexity (17% for complex vs. 11% for non-complex, *p* = 0.367). However, non-complex aneurysms demonstrated a non-significantly higher rate of high DWI burden than complex aneurysms (16% vs. 8%, *p* = 0.277). This was driven by the choice of technique within the non-complex aneurysm group, where complex techniques resulted in double the rate of high DWI burden compared to non-complex techniques (23% vs. 11%, *p* = 0.147). Analysis of individual techniques revealed markedly different risk profiles: BAC (50%), SAC (40%), and Trenza-coiling (31%) were associated with the highest rates, whereas intrasaccular devices (0%), simple coiling (4%), and flow diversion (10%) were associated with the lowest rates of high DWI burden.

## Discussion

Our hypothesis that complex endovascular techniques would achieve superior aneurysm occlusion rates at the cost of increased DWI lesion rates could not be verified. While the overall occlusion rate was high (85%), we observed substantial variation in DWI lesion rates across individual techniques and devices (42–100%) despite relatively uniform occlusion outcomes (70–100%). Although the differences in DWI lesion rates between treatment groups did not reach statistical significance, this may be due to a type II error. This discrepancy between DWI lesion burden but relatively stable occlusion efficacy suggests that technique selection should be emphasized, particularly for non-complex aneurysms where simpler approaches offer superior risk–benefit profiles. A key finding was the seemingly paradoxical observation that non-complex aneurysms were associated with a higher rate of high ischemic burden (≥ 6 DWI lesions) than complex aneurysms (16% vs. 8%, *p* = 0.277). This was due to the use of complex endovascular techniques to treat these simpler aneurysms, which nearly doubled the risk of a high lesion burden compared to non-complex approaches (23% vs. 11%). Applying technically demanding treatments to straightforward anatomy may unnecessarily increase ischemic risk and risk for neurological deficits. This finding further strengthens the view that non-complex treatment offers a better risk–benefit profile than complex treatment for non-complex aneurysms.

Our findings align with the existing literature, suggesting that procedures with minimal parent vessel hardware carry lower thromboembolic risk [1]. The overall DWI rate of 63% is consistent with recent studies reporting 47–59% [5, 15, 26], with our slightly higher rate likely reflecting the higher rate of complex treatments in our cohort (55% vs. 11% in [15]) and MRI acquisition at 2 days versus up to 5 days [15] but similar to the multicenter French ACET study (overall DWI rate 59%) where the MRI delays was 1 day and 41% of aneurysms were treated using a complex technique [26]. Our technique-specific DWI rates largely mirror published data: flow diversion (our 63% vs. ACET 62%), SAC (our 90% vs. ACET 48%), and simple coiling (our 42% vs. ACET 47%) [15]. In comparison to a recent French multicenter study, our results were near to identical or showed similar trajectories of any DWI lesion or ≥ 6 DWI lesions for coiling (any lesion: our 42% vs. ACET 41%; ≥ 6 lesions: our 4% vs. ACET 12%), BAC (any lesion: our 100% vs. ACET 76%; ≥ 6 lesions: our 50% vs. ACET 44%), SAC (any lesion: 90% vs. 90%; ≥ 6 lesions our 40% vs. ACET 50%), intrasaccular device (any lesion: our 64% vs. ACET 47%; ≥ 6 lesions our 0% vs. ACET 19%), but in our cohort the rate of DWI lesions after flow diversion was notably smaller (any lesion: our 63% vs. ACET 95%; ≥ 6 lesions our 10% vs. ACET 38%) [26]. Although the type of flow diverters might differ, it is not likely that this has a major effect on the seen DWI lesion load as, stent coating and surface modifications does not seem to reduce the DWI lesion load [39].

Specific technique and device analysis revealed distinct risk–benefit profiles. Simple coiling demonstrated optimal outcomes (91% occlusion, 42% DWI, *n* = 42), supporting its use as first-line therapy when anatomically feasible. Intrasaccular devices achieved excellent occlusion (100%) with moderate DWI rates (any lesion 64%, ≥ 6 lesions 0%, *n* = 14), justifying their use for wide-necked aneurysms unsuitable for simple coiling. Previously, intrasaccular devices, like the Woven EndoBridge (WEB), have shown high occlusion rates (80–100%) in wide-necked aneurysms, with DWI incidences of 33–89%, aligning with our results [28]. The high occlusion rate seen with intrasaccular devices may be partly attributable to the small median aneurysm size (4.7 mm) in this treatment group, as lower occlusion rates are common for larger aneurysms [23]. Flow diversion, predominantly used for ICA aneurysms, achieved occlusion rates (82%) consistent with published meta-analyses (70–92%) while demonstrating DWI rates of 63%, reflecting the known DWI lesion risk [21]. Trenza-assisted coiling (classified as a non-complex technique) yielded 81% occlusion, comparable to the 83–90% reported in early studies [29, 38], with our study providing the first prospective DWI data for this technique (69% DWI lesion rate). While this ischemic burden for Trenza-assisted coiling was higher than for other simple techniques, the Trenza device is specifically intended for medium-to-large wide neck bifurcation aneurysms as a simpler alternative to complex techniques like BAC, SAC and flow diversion. Especially flow diversion for bifurcation aneurysms has shown to display high rates of complications (22–45% [10, 20, 25]) with occlusion rates of only 69% [20]. Thus, Trenza-assisted coiling seems to be a viable option to the more complex endovascular treatments for bifurcation aneurysms. Yet, especially for MCA-bifurcation aneurysms, microsurgical clipping still yields the most favorable risk–benefit profile [30]. Notably, some of the differences noted between techniques and devices are inherently affected by variations in aneurysm characteristics, as larger and wide-necked aneurysms were more frequently treated with techniques such as flow diversion or Trenza-coiling compared to simple coiling. This may contribute to higher DWI lesion rates due to increased parent vessel manipulation or greater metallic surface area [15, 16, 29].

It should be mentioned that there were three cases of death, one for a procedure-related complication during simple coiling of a non-complex aneurysm, two after flow diversion of complex aneurysms, where one complex large vertebral artery aneurysm ruptured 2 days after treatment and patient with a large complex basilar tip aneurysm died of non-aneurysm related causes 5 months after the treatment.

The wide variation in DWI burden across techniques and devices (42–100%) indicates that ischemic risk should be considered during treatment planning, particularly given the increased risk for neurological deficits. Although population-based studies associate spontaneous silent infarcts with long-term cognitive decline [3, 35, 36], the applicability of these findings to procedurally induced DWI lesions remains to be established. While the high rates of any DWI lesion for SAC (90%) and BAC (100%) warrant careful consideration, sample sizes for these techniques were limited. Moreover, 2 of 10 SAC cases were unplanned bailout procedures for coil prolapse performed without preprocedural DAPT, which may have inflated the DWI rate in this subgroup. Still, similar DWI rates after SAC and BAC was also noticed in the French multicenter study [26]. Overall, our results suggest that treatment algorithms should incorporate both aneurysm complexity and technique-specific ischemic risk to optimize outcomes beyond occlusion alone. Furthermore, the economic implications of these findings warrant consideration. Complex endovascular techniques are associated with substantially higher costs than non-complex approaches [34]. For non-complex aneurysms, complex techniques offered no improvement in occlusion outcomes and no reduction in DWI lesion rates. Given that no clear benefit was observed, the routine use of more expensive complex treatments for non-complex aneurysms needs justification also from a cost-effectiveness standpoint. However, the cost-effectiveness of treatments like flow diversion may not be fully captured by short-term analysis, as potential benefits emerge from lower long-term retreatment rates [14] and in treating specific subsets, such as aneurysms larger than 12 mm [11].

Strategies to reduce procedural DWI lesion burden include favoring the simplest effective technique for non-complex aneurysms, optimizing periprocedural antiplatelet therapy with platelet reactivity monitoring [19], and minimizing intraprocedural catheter and wire manipulation [16]. Although surface-modified devices represent a theoretically appealing approach, current evidence does not support a reduction in DWI lesion rates with coated flow diverters [39].

## Limitations

The study has some limitations. First, the non-randomized design precludes definitive causal inferences between treatment modalities and risk for MRI-DWI lesions. However, given the numerous aneurysm and patient variables affecting treatment selection and outcomes, true randomization would be challenging and require very large sample sizes. Thus, prospective real-world data with objective outcome assessment may represent the most feasible approach in understanding treatment outcomes in clinical practice. Second, conducted at a single, high-volume academic neurovascular center, the findings may not generalize to other settings. Third, the 6-month follow-up duration precludes assessment of long-term occlusion rates, which may improve over time, particularly for flow diversion [4, 24]. Fourth, inherent differences in aneurysm characteristics (e.g., larger aneurysms treated with flow diversion or Trenza-coiling) confound direct comparisons between techniques, reflecting clinical practice but limiting statistical control. Fifth, small sample sizes for BAC (*n* = 4) and SAC (*n* = 10) result in less precise estimates for these techniques. Sixth, MRI-DWI lesions were assessed at a single early timepoint (median 2 days), preventing us from evaluating their evolution. Seventh, the quadrant analysis, while useful for visualization, relies on mean-based thresholds that may oversimplify the continuous relationship between occlusion, ischemia, and aneurysm characteristics. Finally, we did not assess neurocognitive outcomes, limiting our ability to directly link DWI lesions to all clinical consequences. Moreover, the long-term significance of procedurally induced DWI lesions remains unclear, as existing evidence on the cognitive impact of silent brain infarcts derives from population-based studies of spontaneous lesions, which may not be directly comparable.

## Conclusion

The ischemic burden in endovascular treatment appears driven by the specific technique chosen, rather than by broad categorizations of procedural complexity. For non-complex aneurysms, complex techniques offered no occlusion benefit while increasing ischemic risk. Systematic pre-procedural aneurysm complexity classification should guide treatment, prioritizing the simplest effective technique. Future work should focus on mitigating technique-specific risks when treating truly complex aneurysms.

## Supplementary Information

Below is the link to the electronic supplementary material.

ESM 1 Supplementary Material 1 (DOCX 31.7 KB)

## Data Availability

Researcher-initiated data sharing is not possible due to the Finnish Secondary Use Act (552/2019). Thus, all requests to process data for purposes permitted by the Secondary Use Act are given based on an official decision made by FINDATA (https://findata.fi/en/).

## Declarations

### Study registration

The study is registered at clinicaltrials.gov (NCT06147102).
