# Supplementary material for: Technique and device specific diffusion-weighted imaging detected ischemic lesions and occlusion outcomes in endovascular treatment of unruptured aneurysms
Source: Acta Neurochir (Wien). 2026 Jun 4;168(1):182. doi: 10.1007/s00701-026-06934-z (PMC13427851; doi:10.1007/s00701-026-06934-z)
Supplement: Supplementary file 1 — Supplementary Material 1 (DOCX 31.7 KB) [file 701_2026_6934_MOESM1_ESM.docx]

**Supplementary Table 1**. Baseline characteristics and outcomes for the different endovascular treatment techniques.

| Variable | Non-complex treatment | | | Complex treatment | | | P-Value |
| --- | --- | --- | --- | --- | --- | --- | --- |
|  | **Coiling**  (n=24) | **Trenza-coiling**  (n=16) | **Intrasaccular device**  (n=14) | **Flow diversion**  (n=51) | **SAC**  (n=10) | **BAC**  (n=4) |  |
| ****Patient baseline characteristics**** | | | | | | | |
| Age (years), median (IQR) | 57 (47-64) | 62.5 (58-66) | 64 (54-64.5) | 56 (47-62) | 55 (53-60) | 66 (51-68) | 0.092 |
| Sex |  |  |  |  |  |  | 0.889 |
| Female | 17 (71) | 11 (69) | 10 (71) | 33 (65) | 7 (70) | 4 (100) |  |
| Male | 7 (29) | 5 (31) | 4 (29) | 18 (35) | 3 (30) | 0 (0) |  |
| BMI, median (IQR) | 26 (23-31) | 27 (24-29) | 28 (23-30) | 26 (24-29) | 27 (23-29) | 34 (20-37) | 0.956 |
| Hypertension |  |  |  |  |  |  | 0.766 |
| No | 12 (50) | 5 (31) | 5 (36) | 17 (33) | 4 (40) | 2 (50) |  |
| Yes | 12 (50) | 11 (69) | 9 (64) | 34 (67) | 6 (60) | 2 (50) |  |
| Systolic BP, median (IQR) | 136 (127-145) | 140.5 (126-168) | 148 (125-154) | 136 (128-153) | 145.5 (139-163) | 151.5 (143-160) | 0.329 |
| Diastolic BP, median (IQR) | 89 (84-98) | 90.5 (83-101) | 88 (83.5-101) | 87 (81-98) | 92.5 (83.5-99) | 84.5 (78-93) | 0.844 |
| Diabetes mellitus |  |  |  |  |  |  | 0.903 |
| No | 23 (96) | 14 (88) | 13 (93) | 47 (92) | 10 (100) | 4 (100) |  |
| Yes | 1 (4) | 2 (12) | 1 (7) | 4 (8) | 0 (0) | 0 (0) |  |
| Previous SAH | 4 (17) | 1 (6) | 2 (14) | 12 (24) | 4 (40) | 1 (25) | 0.363 |
| Smoking status |  |  |  |  |  |  | 0.027 |
| Never | 8 (33) | 9 (56) | 3 (21) | 26 (51) | 0 (0) | 1 (25) |  |
| Current smoker | 7 (29) | 3 (19) | 7 (50) | 14 (27) | 5 (50) | 3 (75) |  |
| Previous smoker | 9 (38) | 4 (25) | 4 (29) | 11 (22) | 5 (50) | 0 (0) |  |
| ****Aneurysm characteristics**** | | | | | | | |
| Target aneurysm is a retreatment | 0 (0) | 0 (0) | 0 (0) | 11 (22) | 6 (60) | 0 (0) | <0.001 |
| Modality of primary treatment |  |  |  |  |  |  |  |
| Endovascular | 0 (0) | 0 (0) | 0 (0) | 11 (22) | 4 (40) | 0 (0) | 0.110 |
| Surgical | 0 (0) | 0 (0) | 0 (0) | 0 (0) | 2 (20) | 0 (0) |  |
| Aneurysm location |  |  |  |  |  |  | <0.001 |
| ICA segments | 7 (29) | 2 (13) | 3 (21) | 38 (75) | 1 (10) | 4 (100) |  |
| Anterior cerebral artery | 13 (54) | 8 (50) | 8 (57) | 4 (8) | 7 (70) | 0 (0) |  |
| Middle cerebral artery | 2 (8) | 1 (6) | 1 (7) | 1 (2) | 0 (0) | 0 (0) |  |
| Posterior circulation | 2 (8) | 5 (31) | 2 (14) | 8 (16) | 2 (20) | 0 (0) |  |
| Complex aneurysm† | 1 (4) | 0 (0) | 0 (0) | 21 (41) | 4 (40) | 0 (0) | <0.001 |
| Aneurysm largest diameter (mm), median (IQR) | 4.1 (3.1-5.7) | 8.7 (7.9-10.0) | 4.7 (4.3-5.1) | 7.6 (4.9-16.8) | 5.5 (3.2-6.4) | 4.6 (3.5-5.8) | <0.001 |
| Dome-to-neck ratio, median (IQR) | 1.5 (1.2-1.8) | 1.6 (1.3-2.0) | 1.4 (1.2-1.6) | 1.5 (1.1-1.9) | 1.2 (0.9-1.5) | 1.4 (1.2-1.6) | 0.278 |
| Aspect ratio, median (IQR) | 1.6 (1.3-2.0) | 1.5 (1.1-1.7) | 1.1 (0.9-1.4) | 1.4 (1.0-2.2) | 0.9 (0.8-1.5) | 1.5 (1.0-2.1) | 0.045 |

Treatment complexity was defined a priori. Complex treatment included flow diversion, stent-assisted coiling, and balloon-assisted coiling; non-complex treatment included simple coiling, Trenza-assisted coiling, and intrasaccular device placement.

*Ruptured or unruptured aneurysm; †Defined as aneurysm ≥25 mm in size, dome-to-neck ratio <1.0, collateral branch arising from the aneurysm sac; ‡Comparison between all non-complex and all complex; ¶Comparison between all individual techniques.

Abbreviations: BMI=body mass index, BP=bloos pressure, IQR=interquartile range, mm=millimeters, mo=month, SAH=subarachnoid hemorrhage.

**Supplementary Table 2**. Treatment characteristics and outcomes for the different endovascular treatment techniques.

| Variable | Non-complex treatment | | | | Complex treatment | | | | P-Value‡ | P-Value¶ |
| --- | --- | --- | --- | --- | --- | --- | --- | --- | --- | --- |
|  | **All non-complex** (n=54) | **Coiling**  (n=24) | **Trenza-coiling**  (n=16) | **Intrasaccular device**  (n=14) | **All complex** (n=65) | **Flow diversion** (n=51) | **SAC**  (n=10) | **BAC**  (n=4) |  |  |
| **Intervention time*, min (median, IQR)** | 84 (64-97) | 69 (52-87) | 93 (77-123) | 89 (63-98) | 92 (73-117) | 90 (68-113) | 108 (101-155) | 101 (61-133) | 0.031 | <0.001 |
| **Arterial access** |  |  |  |  |  |  |  |  | 0.183 | 0.575 |
| Femoral artery | 47 (87) | 20 (83) | 14 (88) | 13 (93) | 61 (95) | 48 (94) | 10 (100) | 3 (100) |  |  |
| Radial artery | 7 (13) | 4 (17) | 2 (12) | 1 (7) | 3 (5) | 3 (6) | 0 (0) | 0 (0) |  |  |
| **Pre-intervention antiplatelet treatment** |  |  |  |  |  |  |  |  | <0.001 | <0.001 |
| None | 14 (26) | 9 (38) | 1 (6) | 4 (29) | 3 (5) | 0 (0) | 2 (20) | 1 (25) |  |  |
| SAPT | 14 (26) | 9 (38) | 1 (6) | 4 (29) | 1 (1) | 0 (0) | 0 (0) | 1 (25) |  |  |
| DAPT | 26 (48) | 6 (25) | 14 (88) | 6 (43) | 61 (94) | 51 (100) | 8 (80) | 2 (50) |  |  |
| **ARU†, median (IQR)** | 376 (371-389) | 379 (359-387) | 376 (373-512) | 374 (367-386) | 381 (376-468) | 381 (376-474) | 391 (376-453) | 463 (374-552) | 0.155 | 0.654 |
| **PRU†, median (IQR)** | 21 (8-76) | 41 (8-130) | 10 (5-53) | 57 (9-129) | 36 (8-60) | 33 (8-53) | 38 (9-150) | 104 (36-147) | 0.842 | 0.229 |
| **MRI-DWI lesions** |  |  |  |  |  |  |  |  |  |  |
| Any lesion | 30 (56) | 10 (42) | 11 (69) | 9 (64) | 45 (69) | 32 (63) | 9 (90) | 4 (100) | 0.124 | 0.073 |
| ≥6 lesions | 6 (11) | 1 (4) | 5 (31) | 0 (0) | 11 (17) | 5 (10) | 4 (40) | 2 (50) | 0.367 | 0.002 |
| **Occlusion at 6 months§** |  |  |  |  |  |  |  |  | 0.181 | 0.345 |
| Adequate | 45 (90) | 19 (90) | 13 (81) | 13 (100) | 51 (81) | 40 (82) | 7 (70) | 4 (100) |  |  |
| Inadequate | 5 (10) | 2 (10) | 3 (19) | 0 (0) | 12 (19) | 9 (18) | 3 (30) | 0 (0) |  |  |

Treatment complexity was defined a priori. Complex treatment included flow diversion, stent-assisted coiling, and balloon-assisted coiling; non-complex treatment included simple coiling, Trenza-assisted coiling, and intrasaccular device placement.

*Defined as time from puncture to closure

†If measured -- 83 out of 119 patients were tested for ARU and 88 out of 119 patients were tested for PRU using VerifyNow.

‡Comparison between all non-complex and all complex

¶Comparison between all individual techniques

§Calculated for 113 patients with availible 6 month angiographic follow-up

Abbreviations: ARU=ASA response unit; BAC=Balloon-Assisted Coiling, DAPT=Dual Antiplatelet Treatment; PRU=P2Y12 reaction unit; SAPT=Single Antiplatelet Treatment, SAC=Stent-Assisted Coiling
